# Association between cytogenetic alteration and the audiometric profile of individuals with Turner syndrome

**DOI:** 10.1016/j.bjorl.2020.03.005

**Published:** 2020-04-27

**Authors:** Martha Marcela de Matos Bazilio, Adriana Fernandes Duarte dos Santos, Fernanda Gomes de Almeida, Silvana Frota, Marília Guimarães, Márcia Gonçalves Ribeiro

**Affiliations:** a Universidade Federal do Rio de Janeiro (UFRJ), Rio de Janeiro, RJ, Brazil; b Instituto Nacional de Educação de Surdos (INES), Rio de Janeiro, RJ, Brazil

**Keywords:** Hearing loss, Audiometry, Hearing, Hearing disorders, Turner syndrome

## Abstract

**Introduction:**

Turner syndrome is a frequent genetic disorder that affects female individuals and covers a large phenotypic variability. Scientific literature suggests an association between hearing loss and Turner syndrome, but it remains a controversial topic.

**Objective:**

To associate the cytogenetic alteration with the audiometric profile of individuals with Turner syndrome.

**Methods:**

Cross-sectional study, with a hospital-based, convenience sample. Patients diagnosed with Turner syndrome were included and those with difficulty understanding the audiometry and/or other associated syndromes were excluded. The participants were studied with pure tone audiometry.

**Results:**

Of the 65 patients included, 36.9% had X chromosome monosomy and 63.0% had other alterations. Regarding the audiometry, 64.6% had normal thresholds and 35.3% had hearing impairment. Of these, 30.4% had hybrid hearing loss, 26.0% alteration at 6 and/or 8 kHz, 17.3% had conductive hearing loss, 13.0% sensorineural loss and 13.0% had mixed hearing loss. We observed that the mild degree was the most frequent one. There was no statistically significant association between the cytogenetic type of Turner syndrome and the presence or absence of hearing loss, or with the type and degree of hearing loss.

**Conclusion:**

The cytogenetic alteration in Turner syndrome was not associated with the audiometric profile, which showed variability regarding the type and degree of hearing loss.

## Introduction

Turner syndrome (TS) is a genetic, relatively frequent and complex abnormality that affects female individuals and is cytogenetically characterized by sex chromosome monosomy (45X) in mosaicism or not, or by structural alterations. Eventually, the Y chromosome or part of it can be found.^1^, ^2^

TS occurs with a wide variety of anatomical and functional alterations. The clinical picture is variable, with the most common being short stature, immaturity of sexual development, short neck, renal and cardiovascular abnormalities, in addition to a triangular-shaped face, small nose, retrognathism, ogival palate, small jaw and dental malocclusion and some visual alterations (ptosis, strabismus, cataract, nystagmus and myopia), low hair implantation on the nape of the neck and *pectus excavatum.*^3^

Considering the great phenotypic variability and the several comorbidities that can be associated with TS, some studies have suggested an association between hearing loss and TS.^4^, ^5^, ^6^ One of the factors that can influence the type of hearing loss is the karyotype of the individual with TS.^7^

The presence of recurrent chronic otitis media, which contributes to conductive hearing loss, can be explained by the presence of ogival palate in TS, a characteristic that facilitates the occurrence of respiratory disorders and thus hinders the elimination of secretions, which can result in middle ear infections.^3^

Middle ear alterations have been found at a higher percentage in children with TS than in the population of healthy children, even though this disorder is commonly diagnosed in childhood. The authors point out that congenital anatomical changes of the auditory tube, which may be present in TS, can be one of the most probable causes.^5^ Middle ear alterations found in adult women with TS may be due to the lack of adequate treatment in childhood, even though this type of alteration is expected to have been overcome at this stage, due to the growth of middle ear structures.^4^

In addition to middle ear alterations found in TS, other hearing disorders have also been reported. One of them is early presbycusis, due to estrogen deficiency. Progressive sensorineural hearing loss can also be observed, usually starting at mid-frequencies, which can reach high frequencies with advancing age. This type of loss is related not only to age, but also to the early occurrence in individuals with a monosomy, rather than other types of chromosomal alterations.^6^

Knowing the relevance that the sense of hearing has in the lives of individuals and its important role regarding aspects of learning, oral communication and language development,^8^ it becomes essential to carry out studies on the auditory system integrity and function in TS, since for some genetic syndromes, such as Pendred, Alport, and Wanderburg, sensorineural hearing loss is recognized as one of its characteristics.^9^, ^10^ However, in TS, the varied auditory patterns identified, and their possible causes still remain a controversial topic, as well as the influence of the cytogenetic pattern on the hearing loss.^11^

The aim of this study was to associate the cytogenetic type to the audiometric profile of patients with TS.

## Methods

A descriptive and cross-sectional study was carried out, after being approved by the research ethics committee of instituto de puericultura e pediatria Martagão Gesteira (IPPMG/UFRJ), under number 1864085. All individuals who agreed to participate signed the free and informed consent form and the term of assent, when necessary.

This was a hospital-based convenience sample consisting of individuals with a cytogenetic diagnosis of TS, aged 9–39 years old, from the medical genetics and pediatric endocrinology services of IPPMG/UFRJ and the endocrinology service of the hospital universitário Clementino Fraga Filho (HCFF/UFRJ).

Participant selection was carried out through evaluation of medical records and subsequent anamnesis. The inclusion criteria included having a diagnosis for TS with numerical or structural karyotype alterations (lymphocyte culture). The exclusion criteria included the coexistence of another genetic condition (another associated syndrome) and/or the impossibility of performing the audiometry due to the lack of understanding of the requested tasks.

The hearing assessments were carried out in the audiology division of the national institute of deaf education (INES, *Instituto Nacional de Educação de Surdos*), which belongs to the ministry of education. The procedures were performed by three trained audiologists, during a single consultation. An Interacoustics audiometer, model AD 229b, was used in the test, which was calibrated annually, according to ISO 1994.

The results of the cytogenetic study were obtained, and the participants were grouped into the following types: (a) Type 1: X chromosome monosomy; (b) Type 2: other cytogenetic alterations, such as structural changes and mosaicism.

A careful otoscopic inspection of the external auditory canal was performed to visualize the tympanic membrane and possible identification of individuals with a foreign body and/or a wax plug, which would prevent the correct attainment of tonal thresholds.^12^ These individuals were referred to the otorhinolaryngologist for the removal of the foreign body and/or ear wax, returning later for the evaluation.

Then, pure tone audiometry was performed, aiming to determine the audiologic thresholds. The procedure was carried out inside an acoustic booth with the use of headphones, where tonal values were assessed by air at frequencies of 250–8000 Hz and by bone conduction at frequencies of 500–4000 Hz. Thresholds were obtained through the descending/ascending technique.^12^

A normal audiometry was considered as the presence of audibility thresholds up to 25 dB in all frequencies.^13^ Therefore, two distinct groups were created, Group 1 (G1) consisting of individuals with normal hearing thresholds, and Group 2 (G2) consisting of individuals with altered hearing thresholds.

Aiming to designate hearing loss by type, the following classifications were used: conductive, mixed; sensorineural, alteration at 6 and/or 8 kHz.^14^ For the cases with differences between the right and left ears, regarding the type of hearing loss, (for instance: sensorineural in the right ear and alteration at 6 and 8 kHz in the left), a group called hybrid hearing loss was created for this study, as we wanted to classify the type of loss per individual and not by ear.

To designate the degree of hearing loss, the World Health Organization classification (WHO, 1997) was used^13^ through the pure tone average obtained by the air conduction thresholds between the frequencies of 500 Hz, 1000 Hz, 2000 Hz and 4000 Hz. For cases of asymmetric hearing loss in relation to the degree, the ear with the worst degree of hearing loss was considered.

A descriptive analysis was performed, with frequency distribution and measures of central tendency and dispersion at the exploratory level, and the Chi-Square test was used to assess the association between the variables: normality and hearing impairment (G1 and G2) with cytogenetic alterations (Type 1 and Type 2, respectively) and Fisher's exact test to calculate the probability of association between the variables type and degree of loss with karyotype. The sample showed a normal distribution, according to the Shapiro–Wilk test. Analysis of variance (ANOVA) was used to study the influence of age on groups G1 and G2. The statistical significance was set at *p* < 0.05.

## Results

Initially, 70 patients were enrolled, with a mean age of 19.4 years (±7.84). Four patients were excluded because they were unable to understand the tasks proposed in the tests, and one patient, for having a genetic comorbidity (Trisomy 13).

Thus, 65 participants were included: the minimum age was 9 years and the maximum was 39 years. The mean age was 19.1 years (±7.1). Twenty-four (36.9%) patients had X-chromosome monosomy (Type 1) and 41 (63.0%) had other alterations (Type 2).

**Figure 1:**
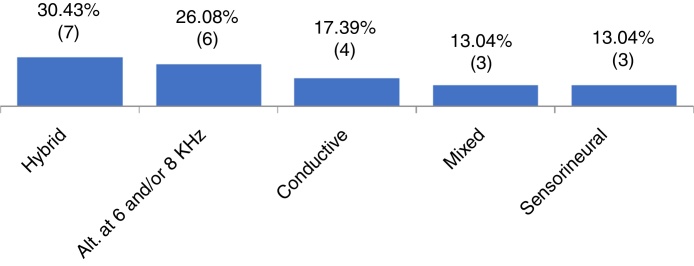
Distribution of patients according to type of hearing loss (Alt, alteration; kHz, kilohertz).

When analyzing the pure tone audiometry responses, we observed that 42 participants (64.61%) had thresholds within the normal range at all evaluated frequencies (G1), whereas 23 (35.3%) had some type of hearing loss (G2). Figure 1 shows the frequency of the types of identified hearing loss.

Regarding the degree of hearing loss, 60% of the sample had mild degree, 20% moderate and 20% had a severe degree. It was not possible to identify the degree of loss in 13 participants, as they had hybrid hearing loss or alteration at 6 and 8 kHz.

No statistically significant association was identified between the cytogenetic type (Type 1 and Type 2) and the audiometry result (G1 and G2) and between the cytogenetic type (Type 1 and Type 2) and the type and degree of hearing loss (Table 1).

**Table 1:** Distribution of patients by cytogenetic alteration and type and degree of hearing loss.

	Type 1	Type 2	*p*-value
*Type of loss*			
Hybrid	1 (14%)	6 (86%)	0.62^a^
Alt at 6 and/or 8 kHz	3 (50%)	3 (50%)	0.27^a^
Conductive	1 (25%)	3 (75%)	1^a^
Mixed	1 (33%)	2 (67%)	1^a^
Sensorineural	0 (0%)	3 (100%)	0.53^a^

*Degree of loss*			
Mild	1 (17%)	5 (83%)	1^a^
Moderate	1 (50%)	1 (50%)	0.46^a^
Severe	0 (0%)	2 (100%)	1^a^

Type 1, monosomy X; Type 2, other alterations; Alt, alteration.

a Fisher's Exact Test.

**Figure 2:**
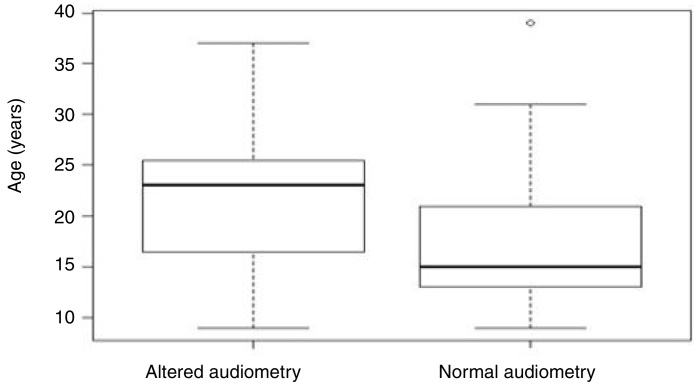
Box-plot graph of mean age comparison (in years) between the normal and altered audiometry groups.

Aiming to analyze the influence of the age factor on G1 and G2, the analysis of variance (ANOVA) test between groups was used, in which the *p*-value obtained was 0.07 and, therefore, not statistically significant (Table 2). However, Figure 2 shows that the individuals in G2 had an older mean age (21.5 years) than those in G1 (17.7 years).

**Table 2:** Influence of age on the variables normal × altered audiometry.

Group	*n*	Mean age in years	SD	Minimum age in years	Maximum age in years	*p*-value
G1	42	17.79	6.81	9	39	0.074^a^
G2	23	21.04	7.35	9	37	0.074^a^

Total	66	18.97	7.13	9	39	

a ANOVA. SD, Standard Deviation; G1, group comprised by individuals with normal audiometry; G2, group comprised by individuals with altered auditory thresholds.

## Discussion

In studies that associate hearing loss and TS, the sample is usually mostly constituted of individuals with 45X, with this cytogenetic type being found in 50% of cases with TS^15^ and being identified as the one most susceptible to hearing loss.^5^, ^7^, ^16^ However, the result of the present study differed from those studies, which may have been due to the sample, in which other types of cytogenetic alterations were more frequent.

It is known that hearing loss may be associated with TS.^3^, ^4^, ^5^ In the present study, when analyzing the distribution of patients with TS according to the audiometric profile (Fig. 1), a wide variety of results were observed. Different types of losses were found in each ear of the same individual (in our study called “hybrid losses”). Although this finding is not very common in clinical practice, it was the most frequent finding in the studied sample. We did not find any reference in the literature that could justify the reason for the high occurrence of hearing loss with different types in the same individual. In this group with TS, the second most frequently observed type of loss was hearing loss at 6 and/or 8 kHz, followed by conductive (17.3%), mixed (13.0%) and sensorineural (13.0%). Other studies indicate different findings regarding the type of hearing loss in the population with TS.^1^, ^5^, ^17^ We suggest that hearing loss in TS is indeed heterogeneous and associated with the types of evaluated samples. In TS studies of which sample size ranged from 51 to 119 individuals, from preschool age to adulthood (73 years), sensorineural loss was identified as the most frequent one,^1^, ^5^ followed by conductive loss.^15^ The variability in audiometric results may be associated with the different age groups assessed and the different criteria used for classifying hearing loss in these studies.

Mild hearing loss was the most frequent and profound hearing loss was not identified in the sample. These results are in agreement with a similar study, which evaluated 52 individuals with TS, aged between 7 and 37 years old, which observed that the participants with hearing loss had a higher frequency of mild, moderate and moderately severe hearing loss, in decreasing order, whereas none had a profound degree of loss.^17^ We believe this finding may be related to the fact that the sample is under 40 years old, considering that hearing loss in TS may be progressive^18^ and that individuals with TS need regular audiological monitoring, as new hearing problems can arise throughout life.^19^

As a limitation of this study, we can point out the small number of participants. However, studies with larger samples still show different results,^1^, ^5^, ^16^ although the association between hearing loss and TS is recognized.^3^, ^4^, ^5^, ^20^

The importance of pure tone audiometry in the diagnosis of hearing loss is undeniable. Although there are several audiological tests at present, pure tone audiometry is still considered the most relevant stage of audiological evaluation, which allows detecting the presence, type and degree of hearing loss.^21^ Moreover, according to international recommendations, audiometry should be performed every five years in adult individuals with TS and every three years in children, regardless of the type of karyotype or the result of the initial hearing assessment.^22^ Therefore, we emphasize the importance of regular audiological monitoring in individuals with TS, aiming to provide adequate treatment and minimize long-term impairment, in addition to the early identification of this comorbidity.

## Conclusion

The type of cytogenetic alteration did not influence whether the individual had or did not have hearing loss, the type and degree of loss. The audiometric profile in TS was varied, with normal hearing function being the most frequent one. Among the hearing alterations identified, there was great variability regarding the type and degree of hearing loss.

## Conflicts of interest

The authors declare no conflicts of interest.

## References

[1] Barreñas M.L., Landin-Wilhelmsen K., Hanson C. (2000). Ear and hearing in relation to genotype and growth in Turner syndrome. Hear Res.

[2] Oliveira R.M.R., Verreschi I.T.N., Lipay M.V.N., Eça L.P., Guedes A.D., Bianco B. (2009). Y chromosome in Turner syndrome: review of the literature. São Paulo Med J.

[3] Mandelli S.A., Abramides D.V.M. (2011). Manifestações clínicas e fonoaudiológicas na síndrome de Turner: estudo bibliográfico. Rev CEFAC.

[4] Hulcrantz M., Simonoska R., Stenberg A.E. (2006). Estrogen and hearing: a summary of recent investigations. Acta Otolaryngol.

[5] Gawron W., Wikiera B., Rostkowska-Nadolska B., Orendorz-Fraczkowska K., Noczynska A. (2008). Evaluation of hearing organ in patients with Turner syndrome. Int J Pediatr Otorhinolaryngol.

[6] Fish J.H., Schwentner I., Schmutzhard J., Abraham I., Ciorba A., Martini A. (2009). Morphology studies of the human fetal cochlea in Turner syndrome. Ear Hear.

[7] Morimoto N., Tanaka T., Taiji H., Horikawa R., Naiki H., Morimoto Y. (2006). Hearing loss in Turner syndrome. J Pediatr.

[8] Neto R.B., Costa O.A., Próteses Auditivas cirurgicamente implantáveis de orelha média, Bevilacqua M.C., Martinez M.A.N., Balen S.A., Pupo A.C., Reis A.C.M., Frota S. (2012). Tratado de Audiologia.

[9] Godinho R., Keogh I., Eavey R. (2003). Perda auditiva genética. Rev Bras Otorrinolaringol.

[10] Silva L.A.F., Kim C.A., Matas C.G. (2018). Characteristics of auditory evaluation in Williams syndrome: a systematic review. Codas.

[11] Alves C., Oliveira C.S. (2014). Hearing loss among patients with Turner's syndrome: literature review. Braz J Otorhinolaryngol.

[12] Frota S., Frota S. (2003). Fundamentos em Fonoaudiologia – Audiologia.

[13] WHO: World Health Organization (2020). http://www.who.int/pbd/deafness/hearing_impairment_grades/en/.

[14] Marzuki N.S., Anggaratri H.W., Suciati L.P., Ambarwati D.D., Paramayuda C., Kartapradja H. (2011). Diversity of sex chomossome abnormalities in a cohort of 95 Indonesian patients with monosomy X. Mol Cytogenet.

[15] Conselhos Federal e Regional de Fonoaudiologia (2013).

[16] Bois E., Nassar M., Zenaty D., Léger J., Abbeele T.V.D., Teissier N. (2018). Otologic disorders in Turner syndrome. Eur Ann Otorhinolaryngol Head Neck Dis.

[17] Oliveira C.S., Ribeiro F.M., Lago R., Alves C. (2013). Audiological abnormalities in patients with Turner syndrome. Am J Audiol.

[18] Kubba H., Smyth A., Wong S.C., Mason A. (2016). Ear health and hearing suvillance in girls and women with Turner's syndrome: recommendations from Turner's syndrome support society. Clin Otolaryngol.

[19] Kubba H., McAllister K., Hunter K., Mason A. (2019). Annual hearing screening in girls withTurner syndrome: results from the first three years in Glasgow. Int J Pediatr Otorhinolaryngol.

[20] Beckman A., Conway G.S., Cadge B. (2004). Audiological features of Turner's syndrome in adults. Int J Adiol.

[21] Momensohn-Santos T.M., Russo I.C.P., Assayag F.M., Lopes L.Q., Momensohn-Santos T.M., Russo I.C.P. (2007). Prática da Audiologia Clínica.

[22] Gravholt C.H., Andersen N.H., Conway G.S., Dekkers O.M., Geffner M.E., Klein K.O. (2017). Clinical practice guidelines for the care of girls and women with Turner syndrome: proceedings from the 2016 Cincinnati International Turner Syndrome Meeting. Eur J Endocrinol.

